# Remote Sensing-Based Quantification of the Impact of Flash Flooding on the Rice Production: A Case Study over Northeastern Bangladesh

**DOI:** 10.3390/s17102347

**Published:** 2017-10-14

**Authors:** M. Razu Ahmed, Khan Rubayet Rahaman, Aaron Kok, Quazi K. Hassan

**Affiliations:** Department of Geomatics Engineering, University of Calgary, 2500 University Dr. NW, Calgary, AB T2N 1N4, Canada; mohammad.ahmed2@ucalgary.ca (M.R.A.); krrahama@ucalgary.ca (K.R.R.); koka@ucalgary.ca (A.K.)

**Keywords:** Landsat-8, MODIS, normalized difference vegetation index (NDVI), multi-temporal data, *boro* rice

## Abstract

The northeastern region of Bangladesh often experiences flash flooding during the pre-harvesting period of the *boro* rice crop, which is the major cereal crop in the country. In this study, our objective was to delineate the impact of the 2017 flash flood (that initiated on 27 March 2017) on *boro* rice using multi-temporal Landsat-8 OLI and MODIS data. Initially, we opted to use Landsat-8 OLI data for mapping the damages; however, during and after the flooding event the acquisition of cloud free images were challenging. Thus, we used this data to map the cultivated *boro* rice acreage considering the planting to mature stages of the crop. Also, in order to map the extent of the damaged *boro* area, we utilized MODIS data as their 16-day composites provided cloud free information. Our results indicated that both the cultivated and damaged *boro* area estimates based on satellite data had strong relationships while compared to the ground-based estimates (i.e., *r*^2^ values approximately 0.92 for both cases, and RMSE of 18,374 and 9380 ha for cultivated and damaged areas, respectively). Finally, we believe that our study would be critical for planning and ensuring food security for the country.

## 1. Introduction

Flash flooding is a recurring natural disturbance over the northeastern part of Bangladesh. This region exhibits a unique landscape that consists of hundreds of “*haors*”, characterized as bowl or saucer-shaped large tectonic depression [1]. These *haors*, collectively known as *haor* basin, is completely flooded between the monsoon and post-monsoon seasons that usually spans between June and November every year [2]. On the other hand, the *haor* basin becomes almost completely dry from December to May when the farmers cultivate the only cereal crop known as *boro* rice [2]. However, excessive local/regional rainfall occurrences during late March to April often trigger flash floods that cause widespread destruction to the *boro* rice, and thus imposes a severe threat to the food supply (also known as food security) nationally. For example, Roy et al. [3] reported that flash flooding hit the region six times in the months between March and April during the period 2000–2017 upon consulting Professor Saiful Islam from Bangladesh University of Engineering and Technology. These dates include: (i) 30 April 2000; (ii) 19 April 2002; (iii) 15 April 2004; (iv) 3 April 2010; (v) 17 April 2016; and (vi) 27 March 2017. Additionally, Biswas [4] analyzed the frequency of early flash flooding that occurred as a function of regional rainfall data available from the Bangladesh Meteorological department between 29 March to 3 April. It revealed that more than 150 mm rainfall during these days could trigger an early flash flooding event. In fact, such events had taken place six times since 1956, with the highest amount of rainfall (i.e., 625 mm) in 2017. Consequently, the river water levels at several key gauge stations had exceeded the site-specific *danger levels*, e.g., 13.20 m, 13.50 m and 11.25 m in the Surma River at Kanaighat, Kushiyara River at Sheola, and Surma River at Sylhet respectively during the first week of April 2017 [5]. The *danger level* is defined for the water level in the river, which has been determined on the basis of annual average flood level at the site of interest. Equal or greater water level in comparison to the *danger level* may trigger the inundation process around the surrounding lands, and potentially cause damages to agricultural crops and settlements [6]. Thus, we opted to map the impact of the 2017 early flash flooding event on the *boro* crop in this study.

In fact, *boro* rice is the main contributor among all other rice types in Bangladesh’s context [7]. For example, it accounted for approximately 55% of the rice production per annum on average between 2011 and 2016 [8]. Also, it is interesting to note that the flash flood-stricken region (i.e., the *haor* basin as mentioned in the previous paragraph) that consists of six districts (i.e., Sylhet, Maulavibazar, Sunamgonj, Hobigonj, Kishoregonj, and Netrokona) in the northeastern part of Bangladesh has produced approximately 15% of the total *boro* production per annum on average between 2011 and 2016 [8]. Thus, any disturbance (such as flash flooding) on the *boro* production potentially impacts the staple food supply significantly for the people at local, regional, and national level. As a result, it is critical to have an efficient method in place to estimate the *boro* acreage/production in a timely fashion. In reality, the Government of Bangladesh (i.e., Ministry of Agriculture) collects and synthesizes rice acreage and production by accumulating statistical data acquired through field visits and interviewing the farmers [9]. However, this process is considered traditional in nature and is time consuming, expensive, labor-intensive, and the information is only available at certain times. In order to address these concerns, one of the efficient, cost-effective, and viable methods is to employ multiple and/or multi-temporal satellite-based remote sensing approach to map rice acreage/production under normal [10,11,12,13] and disastrous conditions [14,15,16,17,18]. In the event of such damage mapping induced by flooding, we could broadly categorize the literature into two classes: radar and optical remote sensing-based approaches. Some of the example case studies where the researchers used primarily radar images are as follows:

- Chowdhury and Hassan [19] employed four RADARSAT-1 Synthetic Aperture Radar (SAR) images acquired before, during, and after the flooding period to delineate the damages on the rice over southwest Bangladesh in 2000;
- Haldar et al. [15] analysed multi-temporal SAR images to generate a rice mask and then to quantify the impact of the flooding in relation to the Phailin cyclone over Odisha, India in 2013;
- Waisurasingha et al. [16] combined a RADARSAT-1 SAR image acquired at the peak of the flooding season, high resolution digital elevation model, and water level at certain gauge stations in order to generate a flood depth map over the lower Chi River floodplain, Thailand in 2001. They used such a depth map in conjunction with a Landsat-7 Enhanced Thematic Mapper Plus (ETM+) derived damaged and non-damaged rice map to quantify the threshold of the flooding depth in relation to the rice damage.
- Lee and Lee [20] acquired four RADARSAT-1 SAR images over pre-, during-, and post-flooding period; and produced three rice classes, such as not-flooded, recovered and not-recovered after flooding. They used a Landsat-5 Thematic Mapper (TM) dataset acquired prior to the flooding season to generate a land use/land cover map; which was then used to validate the SAR-derived damage map.

In reality, the use of the SAR images provides unique solutions in mapping the rice damage as a result of flooding. This is due to the fact that the radar satellites are capable of acquiring images under any atmospheric condition, which is especially useful when considering climatological attributes such as high cloud density in the lower atmosphere and rainfall correlated with flooding events. Despite this, there are two limiting factors, such as (i) the revisit frequencies of these satellites are usually longer (i.e., 24 days for RADARSAT platform), (ii) the images are quite expensive for the rice growing countries that are mostly developing economies.

In order to address the limitations of the radar platforms, optical remote sensing satellites can be used where some of them are available in the public domain at free of cost with reasonable high temporal resolution (such as Landsat with 16-days, and Moderate Resolution Imaging Spectroradiometer, i.e., MODIS at daily-scale in particular). Some of the example cases include:

- Dao and Liou [17] mapped the damage rice over central region in Cambodia in 2013 in three steps. Firstly, they delineated the flooded area using two Landsat-8 images acquired pre- and post-flooding stages. Secondly, they generated the spatial extent of rice cultivation using the time-series of MODIS-based vegetation indices. Finally, they produced the extent of the rice damage upon incorporating the first two steps.
- Kwak et al. [18] proposed a framework consisting of three steps for mapping nationwide rice damages, and implemented over Bangladesh in 2007. Firstly, they employed MODIS based water index to map the flood extent. Secondly, they combined MODIS based vegetation indices, Global Land Cover Dataset by National Mapping Organizations [18], and Global Map of Irrigation Areas (GMIA ver.5) [18] in order to generate the spatial extent of rice. Finally, they created rice damage map by combining the earlier two steps.
- Chohan et al. [21] employed three Landsat-8 images acquired during the pre- and post-flooding season in 2014, and pre-flooding season in 2015 in order to quantify the flood-induced damages on the agricultural crops including rice over the Chenab River floodplain in Hafizabad, Punjab, Pakistan. They applied both supervised classification and soil adjusted vegetation index in such damage assessments.
- Memon et al. [22] studied a set of MODIS derived water indexes to delineate the extent of inundation and its associated damages on various land use/land cover types including agricultural crops over several provinces in Pakistan in 2012. They evaluated these indices against the Landsat-7 ETM+ derived ones.

Upon going through the literature, we decided to use optical remote sensing images despite their limitation in viewing the earth surface in the presence of cloud cover. Thus, the overall goal of this paper was to employ such optical remote sensing images to assess the impact of flooding on the *boro* rice production in the flash flood prone districts of Bangladesh, which were augmented by three specific objectives. Firstly, we opted to use multi-temporal Landsat-8 Operational Land Image (OLI) images obtained during the dominant dry season (spanning between December and March, when the sky would be cloud-free for most of the time) in order to estimate the *boro* rice acreage, and its validation with the estimates available from the Department of Agricultural Extension (DAE) of the Bangladesh Government. In fact, our earlier study (i.e., [23]) demonstrated that MODIS images acquired during the similar time-period (i.e., January to March) over all of Bangladesh would be capable of estimating the *boro* rice acreage. Secondly, we planned to use 16-day composite of MODIS derived normalized difference vegetation index (NDVI) between December 2016 and April 2017 in order to estimate the damages on the *boro* rice in particular, and its validation using the reported damage estimates by the Department of Disaster Management (DDM) of the Bangladesh Government. It was the case that there were no cloud-free Landsat 8 images available for the area during the month of April 2017. Finally, we produced the spatial extent of survived/damaged *boro* by combining the outcomes from Landsat-8 and MODIS data to aid the better comprehension of the impact at landscape level.

## 2. Study Area and Data Requirements

### 2.1. General Description of the Study Area

**Figure 1:**
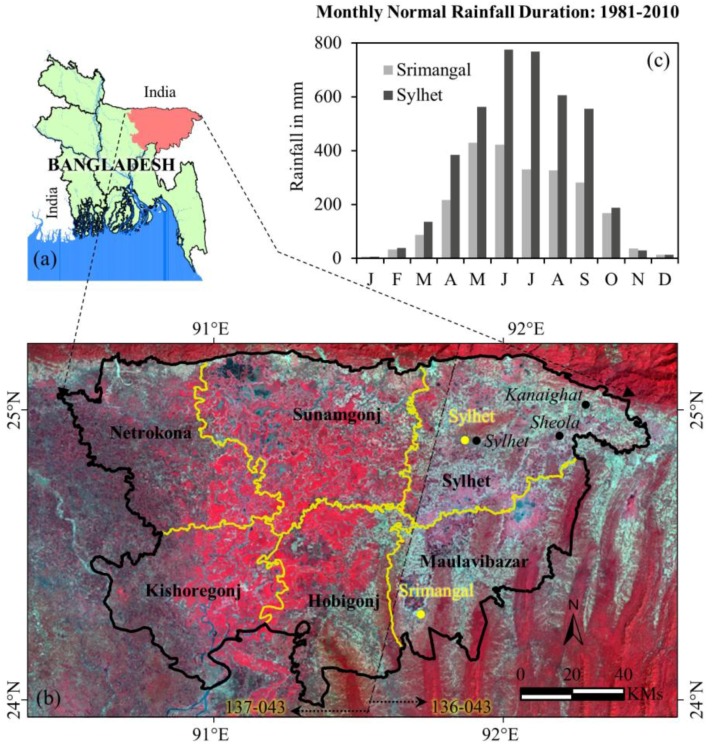
Map showing the spatial extent of the study area in Bangladeshi context (**a**,**b**), where the black and yellow polygons in (**b**) show the boundaries of the study area and individual district respectively. Additionally, the monthly normal rainfall distributions during the period 1981–2010 at Sylhet and Srimangal weather stations [yellow points in (**b**)] are shown in (**c**); and locations of water level measured above *danger level* during the flooding event are shown as black points in (**b**).

We considered six northeastern districts of Bangladesh as our study area, as shown in Figure 1. The study area lies between 23°58′ to 25°12′ North latitude and 90°27′ to 90°30′ East longitude covering an area of 17,800 km^2^. The central portion of the study area falls within the extent of the depressed zone known as *haor* basin. This basin occupies approximately 24,500 km^2^ [24] that is situated within our study area and beyond. It is also bounded by the hill ranges of India, with Meghalaya to the north, Tripura and Mizoram to the south, and Assam and Manipur to the east [25]. As mentioned in the first paragraph of the ‘Introduction’ section, the *haor* basin primarily supports the cultivation of *boro* rice through the year. Beyond the *haor* basin, the eastern portions of the study area consisting of Sylhet, Maulavibazar and Habigonj districts are mostly highlands or hillocks, where the major land use/ land cover types are tea garden, natural and planted forests, and shrubs.

In terms of climate regimes, the study area observes sub-tropical conditions with three dominant seasons such as, (i) dry winter (December to February); (ii) pre-monsoon hot summer (March to May); and (iii) the rainy monsoon season (June to October) [23]. This region experiences the coldest temperature (i.e., in the range 10–12.9 °C) in January and the hottest temperature (~32 °C) in April [26]. The area receives a significantly higher amount of rainfall ranged between 3280 and 4780 mm in comparison to the national average (i.e., 1600–2300 mm) [27]. Such higher amounts of rainfall are demonstrated at two weather stations (i.e., Srimangal and Sylhet) in Figure 1 in terms of normal rainfall regimes over the period 1981–2010. Also, it reveals that small amounts of rainfall usually occur between January to March. In April, the rainfall starts to increase, and this is crucial to support the growth and sustainability of the *boro* crop.

### 2.2. Data Used and Its Pre-Processing

In this study, we evaluated the freely available Landsat-8 OLI images in the form of both surface reflectance and NDVI at 30 m spatial resolution freely available from United States Geological Survey (USGS) websites over the study area between December 2016 and April 2017. Note that these images were acquired with a combination of path-row 136-043 and 137-043 in the right and left portions of the study area, respectively, as shown in Figure 1. This process led us to determine two sets of cloud-free images acquired at: (i) 16 December 2016, 17 January 2017, 18 February 2017, and 22 March 2017 for the path-row 137-043; and (ii) 9 December 2016, 10 January 2017, 11 February 2017, and 15 March 2017 for the path-row 136-043. It also revealed that there were no cloud-free images available in April 2017 over the study area. Thus, we employed these images acquired between December 2016 and March 2017 (i.e., the pre-flash flooding time period) to map the areas under *boro* cultivation, where this period coincided with the *boro* growing conditions associated with the plantation to mature stages.

As there were no cloud-free Landsat-8 images available during the month of April 2017, we opted to explore the adaptability of other freely available images from National Aeronautics and Space Administration (NASA). These were the MODIS-derived 16-day composite NDVI images at 250 m spatial resolution (i.e., MOD13Q1 version 006) for both pre- and post-flash flood conditions between December 2016 and April 2017 and onward over the study area in order to assess the damage associated with *boro* rice. It would be worthwhile to mention that MODIS 16-day composite had the advantage of eliminating most of the cloud-contaminated pixels found in a single image. Also, such composite was successfully used for *boro* mapping in other studies [9,14,23]. The dates of the MODIS NDVI composite images were: 18–31 December 2016, 1–16 January 2017, 17 January–1 February 2017, 2–17 February 2017, 18 February–5 March 2017, 6–21 March 2017, 7–22 April 2017, 23 April–8 May 2017, 9–24 May 2017, and 25 May–9 June 2017. Note that we could not use the 22 March–6 April 2017 composite image in the time-series due to the presence of thick clouds during the period of interest.

Upon acquiring all the required datasets, we performed the following set of pre-processing steps to both Landsat-8 OLI and MODIS images. Those included: (i) re-projecting the images into Universal Traverse Mercator (UTM) Zone 46 N with World Geodetic System 1984 (WGS84) datum, (ii) clipping the images to the extent of the study area, (iii) identifying cloud contaminated-pixels using “Quality Assessment” information for the MODIS images to exclude them from further analysis, and (iv) generating multi-temporal NDVI images. These images were subsequently used in mapping of *boro* acreage, *boro* damage, and extent of damaged/survived *boro*, which are described in the following section.

Apart from using satellite images acquired through Landsat-8 and MODIS, we also used a very high-resolution image source (i.e., Google Earth) for the identification and verification of the classified *boro* fields. Note that Google Earth had been used in several studies for the identification and verification of land cover such as, water, rice crop fields and others [9,14,23]. In addition, we used agricultural statistical ground information for (i) *boro* rice cultivated areas (i.e., pre-flash flood conditions) freely available from DAE as reported in [3]; and (ii) *boro* damage/survive assessment (i.e., crop damage due to the flash flooding) from freely available from DDM as reported in Nirapad [28]. The DAE collects the agricultural statistical data by conducting field surveys and interviewing farmers. The estimation of cultivated rice areas is performed through annual and/or seasonal surveys in the ground. Field staff record the survey data several times over the growing seasons, which are further checked and processed by the regional statistical officers for generating the national agricultural statistical data [14].

## 3. Methods

**Figure 2:**
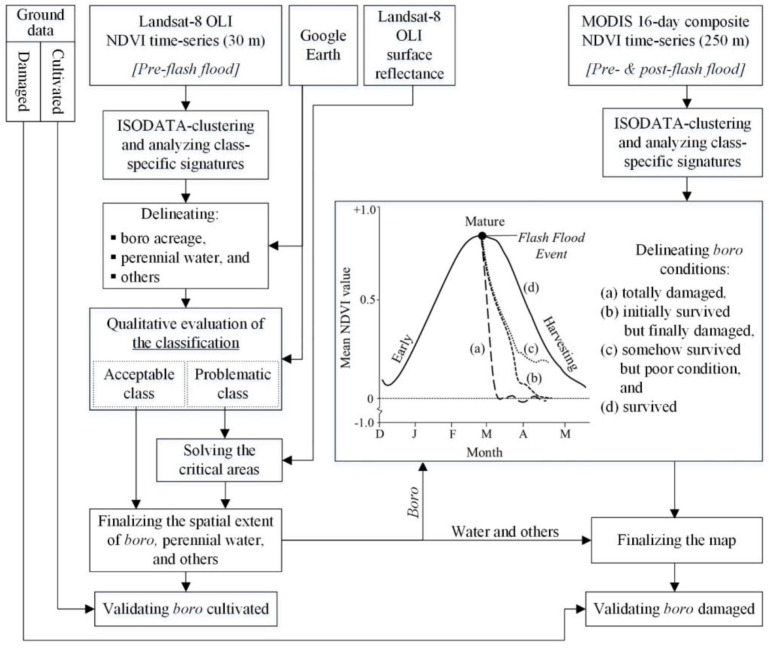
Schematic diagram of the proposed methods and their validation.

Figure 2 shows the schematic diagram of the proposed methods. It consisted of three major components: (i) mapping of cultivated *boro* acreage before the flash-flood using Landsat-8 OLI images, (ii) mapping of *boro* damage after flash-flood event using MODIS images, and (iii) determining the extent of damaged/survived *boro* in the study area upon combining the outcomes from the first two components. These are briefly described in the following sub-sections.

### 3.1. Mapping of Cultivated Boro Rice Acreage from Landsat-8 OLI Images

In order to map the cultivated *boro* rice acreage using Landsat-8 OLI images during the pre-flash flood, we performed a set of steps. Firstly, we applied a clustering algorithm, i.e., iterative self-organizing data analysis technique (ISODATA) over the two sets (i.e., 136-043 and 137-043) of multi-temporal NDVI images (i.e., NDVI time-series). We adopted the ISODATA clustering algorithm because of its ability to statistically assign every pixel of an image to generate classes based on spectral similarities [29], to define multiple classes using inherent characteristics in the time-series (i.e., multi-temporal), and to classify larger extent of heterogeneous landscapes (includes, various vegetation types) [30]. This clustering technique generated 50 classes from each set of time-series with convergence threshold of 0.995 upon keeping an infinite number of iterations. We intended to generate three major land cover classes: (i) *boro*, (ii) perennial water, and (iii) others by use of temporal signatures for the classes of interest. Note that the open surface water class from the time-series was considered as the perennial water, because these images were acquired during the driest period of the year (see the second paragraph of the section “Study area and data requirements”) [7,31].

In the second step, we evaluated the class-specific temporal signatures in grouping the 50 classes into the intended three land cover classes as mentioned earlier. In order to aid this process, we consulted several other sources, such as: (i) Google Earth image; (ii) surface reflectance-based multi-spectral Landsat-8 bands with various combinations; and (iii) the patterns associated with *boro* rice mapping described in Mosleh and Hassan [9], to identify the patterns of temporal signatures for each land cover class based on their spatial distribution on the ground. At this phase, we observed that few signatures were misclassified as *boro* rice, while comparing with multi-spectral Landsat-8 data acquired on 22 March 2017, which were mixed with perennial water class representing portion of waterbodies. Therefore, we grouped all the *boro* specific-signatures into two quality classes: (i) acceptable, and (ii) problematic.

Upon identifying the problematic areas, we opted to use Landsat-8 based surface reflectance image acquired in the month of March 2017 in the next step. In this case, we examined the surface reflectance values of water for the spectral bands of near infrared (NIR) and red (R) bands. Our analysis revealed that the values of NIR and R bands were less than 0.1871 and 0.068 respectively over the water bodies. Thus, we used these values as thresholds in producing a water class in our model. Finally, we added the acceptable and rectified classes (i.e., converted from *boro* to perennial water class) for generating cultivated *boro* acreage map. We eventually generated two *boro* acreage maps from two the sets of NDVI time-series (i.e., 136-043 and 137-043), and mosaiced those in order to generate a cultivated *boro* rice acreage map of the entire study area.

Finally, we validated the map with the ground data. In this process, we calculated the cultivated *boro* rice acreage over six districts, and compared it with the statistical ground data. We determined the degree of agreements between our mapped and ground-based estimates in two ways: (i) percentage error or deviation at study area-level, and (ii) coefficient of determination (i.e., *r*^2^) from the linear regression analysis, and root mean square error (RMSE) at the six district-levels.

### 3.2. Mapping of Damaged Boro Acreage from MODIS Images

We performed two major steps to map the damaged *boro* due to the flash flood using MODIS images during the pre- and post-flash flood. Firstly, we applied an ISODATA-clustering algorithm to the MODIS-based NDVI time-series. The process generated 50 classes with the same convergence threshold and iterations mentioned in the previous sub-section. In this case, we considered four different conditions associated with *boro* rice, such as: (a) totally damaged, (b) initially survived but finally damaged, (c) somehow survived but poor condition, and (d) survived or not affected.

In the second step, we evaluated the ISODATA-clustering derived classes that fell within the Landsat-8 derived *boro* acreage map (as discussed in Section 3.1). Subsequently, we reviewed each of the class-specific temporal signatures of mean NDVI-values, and associated them with one of the generic four *boro* conditions (see Figure 2 for details). Such generic signatures were generated upon exploiting the NDVI profiles in relation to the *boro* growth stages under normal environmental/climatic conditions as described in Mosleh and Hassan [9]. Note that the flash flood occurred during the ‘mature’ stage of the *boro* rice in our study area, so we therefore assumed that the similarities or deviations of the NDVI signatures compared to the normal growth profile of *boro* after the flash flooding event would represent different *boro* conditions. These included:

- Totally damaged *boro*: sharp drop of NDVI-values to zero or close, which indicated that *boro* crop was totally submerged;
- Initially survived but finally damaged *boro*: gradual declination of NDVI-values to zero or close in the second or third subsequent imaging periods following the flooding event;
- Somehow survived but poor condition *boro*: gradual declination of NDVI-values to lower magnitudes (e.g., around 0.4), and continue with the similar values for rest of the season; and
- Survived or not affected *boro*: the signatures would follow similar patterns with that of normally growing *boro* crop.

Upon defining the four *boro* classes, we merged them with the Landsat-8 derived perennial water and other classes in order to generate the spatial dynamics of the study area.

Finally, we evaluated the estimates of damaged *boro* rice derived from remote sensing data with the ground data available from DDM [28]. In this case, we considered the two classes from the final map (i.e., totally damaged *boro*, and initially survived but finally damaged *boro*) for our estimates of damaged *boro* rice acreage. In the evaluation process, we quantified the degree of agreements between the damaged *boro* and ground-based estimates in two ways: (i) percentage error or deviation at study area-level, and (ii) *r*^2^ from the linear regression analysis, and RMSE at the six district-levels.

## 4. Results

### 4.1. Mapping of Cultivated Boro Rice Acreage from Landsat-8 OLI-Derived NDVI Time-Series

**Figure 3:**
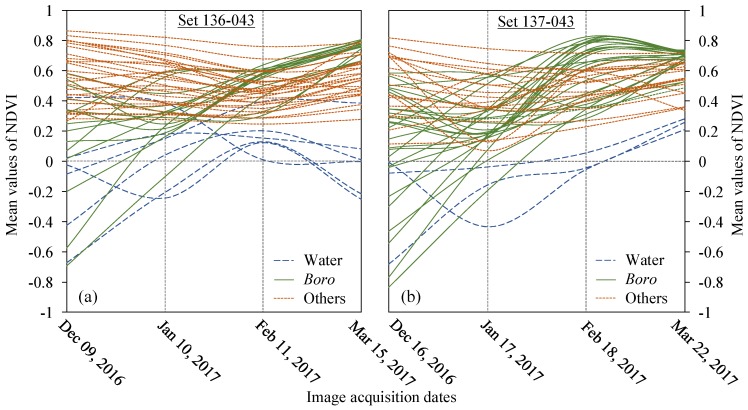
Temporal dynamics of mean NDVI-values of *boro*, water, and others over the planting to mature season using time-series of Landsat-8 OLI data for the path-row: 136-043 (**a**), and 137-043 (**b**).

Figure 3 shows the temporal signatures of Landsat-8 derived NDVI-values between December 2016 and March 2017 (i.e., the pre-flash flooding time). We found that the signatures of our three intended classes (i.e., *boro*, water, and others) were distinctive. For example, the signatures of ‘water (perennial)’ class showed very low mean NDVI-values (i.e., zero or close) for the entire period of the time-series. Note that few signatures showed a little higher mean NDVI-values, which were probably due to the presence of floating or submerged water-weeds, and turbid conditions of the waterbodies. The signatures of the ‘others’ class showed higher mean NDVI-values over the period of the time-series. The *boro* rice exhibited unique characteristics between the initial planting or transplanting stage (i.e., December to February) and the mature stage (i.e., in March). The initial stage showed the lowest mean NDVI-values indicating very low level of biomass, and the mature stage portrayed the highest mean NDVI-values due to it being full grown and flowering (i.e., the maximum level of greenness/biomass). Note that the initial stage showed a wide range of variation in the mean NDVI-values, which were probably due to the variable planting/transplanting times spanning for about three months (i.e., from December to February). Such a lengthy initial stage was due to the gradual recession of water in the *haors*. Nevertheless, the mean NDVI-values showed increasing trends from the initial to mature stages, which were representing the gradual phenological development of the *boro* crop towards the maturity. Our observed temporal dynamics of the signatures in relation to the development stages were found similar with those reported in literature (e.g., [9,14,23]). Additionally, remote sensing-derived signatures of rice crops studied in other countries demonstrated similar dynamics in growing stages [32,33].

**Figure 4:**
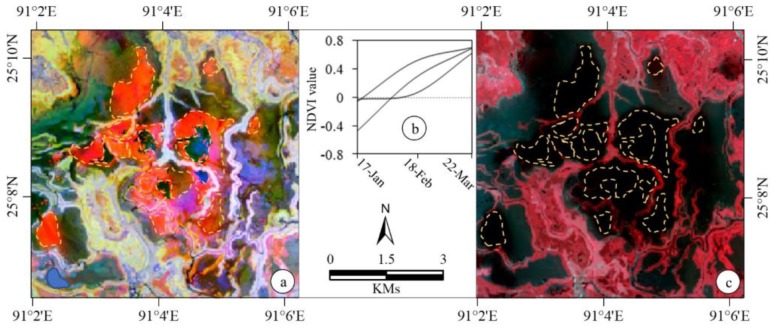
Example of misclassification of the portion of water bodies over north-west portion of the Sunamgonj district as *boro* rice as depicted using yellow dotted polygons on the multi-temporal (acquired on 17 January, 18 February, and 22 March 2017) NDVI images derived from Landsat-8 data (panel(**a**)); its resultant multi-temporal NDVI signature (**b**). Also, these areas were, in fact, potion of waterbodies as shown using yellow dotted polygons on a false colour composite of the multi-spectral Landsat-8 data acquired on 22 March 2017 (**c**). In panel (**a**), redish, black to blueish, and yellowish colours show misclassified water bodies as *boro*, water bodies, and *boro* respectively. On the other hand, black to blueish, and redish colours in panel (**c**) show water boides, and *boro* respectively.

Among the *boro*-specific NDVI-based signatures as shown in Figure 3, some of them resulted in misclassification of perennial water bodies as *boro* rice. Such an example case is shown in Figure 4. In fact, we consulted the multi-spectral Landsat-8 data acquired on 22 March 2017 to address this problem, where the detailed steps were discussed in Section 3.1. Consequently, we demonstrated the effectiveness of using the multi-spectral Landsat-8 data in minimizing the classification errors in the event of only using the NDVI time-series.

**Figure 5:**
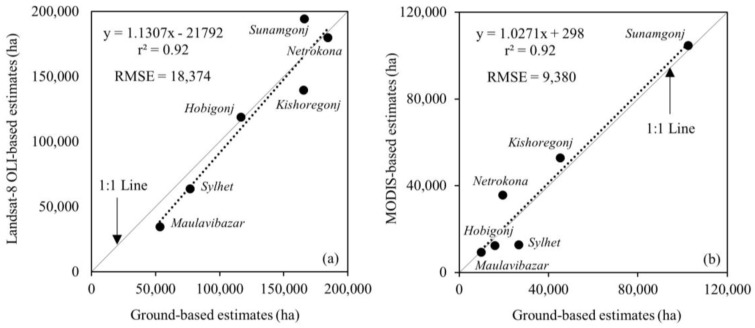
Relations between ground and remote sensing-based estimates of *boro* cultivated (**a**) and damaged (**b**) at six districts, where the dash lines represented the regression lines.

Upon determining both the acceptable *boro* signatures and revisions to the problematic ones, we produced a Landsat-8 based acreage of cultivated *boro* rice prior to the onset of the flash flooding event. Subsequently, we compared them with the ground-based estimates at both study area- and six districts-level. In both cases, we found that the level of agreement was strong, i.e., (i) percentage error of 4% at the study area-level; and (ii) *r*^2^-value of 0.92, and RMSE of 18,374 ha at the six district-level (see Figure 5a).

### 4.2. Mapping of Damaged Boro Rice Acreage from MODIS-Derived NDVI Time-Series

**Figure 6:**
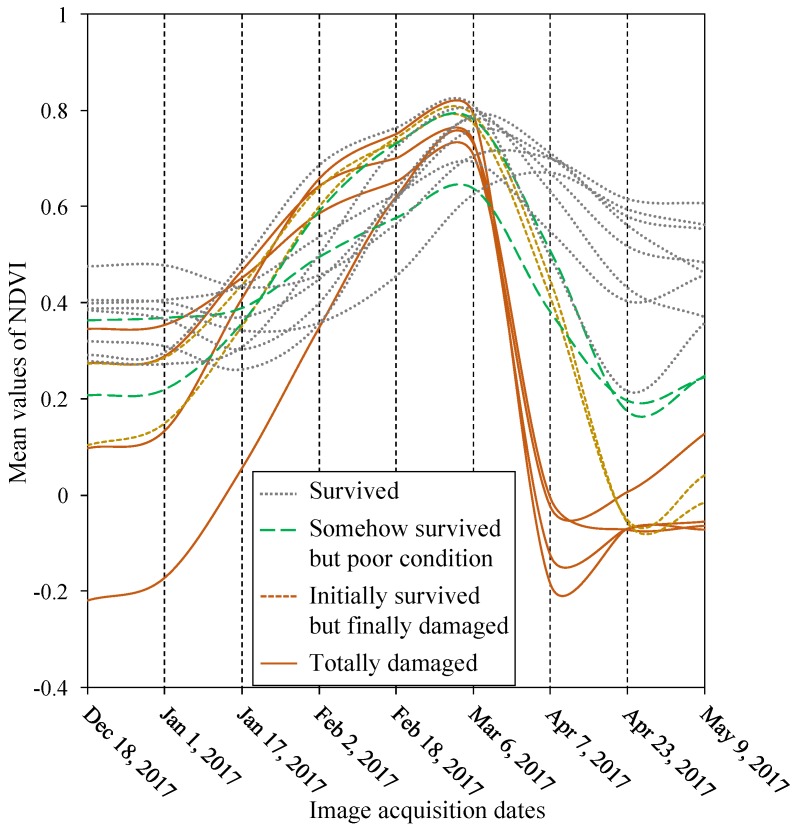
Temporal dynamics of MODIS-derived mean NDVI-values over the planting to harvesting season indicating various *boro* conditions after the flash flooding event.

Figure 6 shows the temporal signatures of MODIS-derived NDVI-values between December 2016 and May 2017 (i.e., the pre- and post-flash flooding times). Upon compiling the *boro* condition-specific signatures and identifying the corresponding spatial locations, we generated a final damage map, where the “initially survived but finally damaged” and “totally damaged” classes were considered as the damage *boro* acreage for the assessment purposes. Such damage estimates were then compared against ground-based estimates. In these cases, we also observed strong relationships between them, such as: (i) percentage of error of −3% at the study area-level, and (ii) *r*^2^-value of 0.92, and RMSE of 9380 ha at the six district-level (see Figure 5b).

### 4.3. Spatial Dynamics of Culivated and Damaged Boro

**Figure 7:**
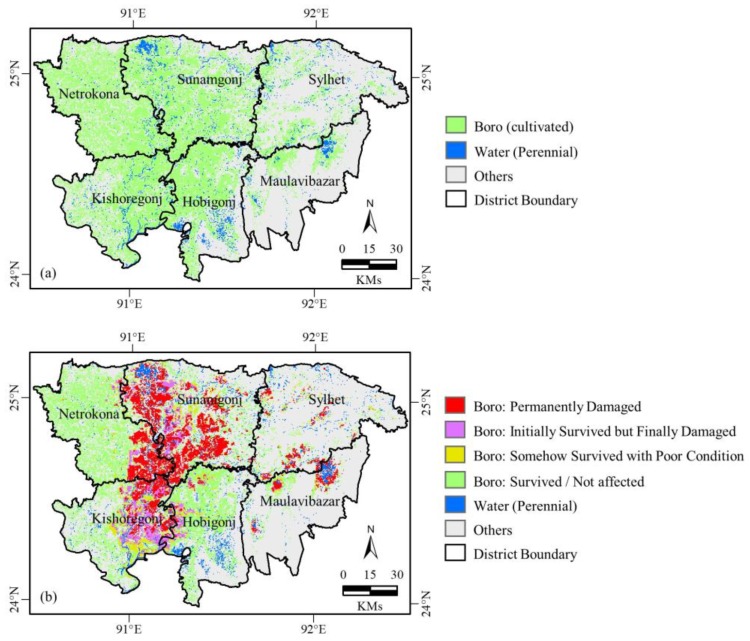
Spatial extent of: (**a**) cultivated *boro* derived from Landsat-8 OLI images during the pre-flash flooding time period, and (**b**) damaged *boro* derived from MODIS images due to the flash flooding event. Also, the cumulative area under all the four *boro* conditions as depicted in panel (**b**) were, in fact, same as the cultivated *boro* rice acreage estimated using Landsat-8 data as shown in panel (**a**).

Figure 7 shows the spatial extent of Landsat-8 derived cultivated *boro* during the pre-flash flooding time period (panel (a)), and MODIS-derived damaged *boro* as a result of the flash flooding event (panel (b)). According to Figure 7a, we found that about 34.5% of the study area was under *boro* rice cultivation. It would be interesting to note that major areas of the districts of Netrokona (i.e., about 63% of the total area), Kishoregonj (i.e., about 56%), Sunamgonj (i.e., about 52%), and Hobigong (i.e., about 46%) were used *boro* cultivation. On the contrary, relatively smaller areas in the districts of Sylhet (i.e., about 19% of the total area) and Hobiganj (i.e., about 13%) were undergone through *boro* cultivation.

In order to determine the extent of damaged *boro*, we investigated its dynamics based on exploiting the MODIS images over the areas that were classified as the cultivated *boro* rice areas by use of Landsat-8 data as shown in panel (a). We found that about 37% of the cultivated *boro* rice was damaged due to the flash flooding. Note that we considered the two conditions of *boro*, i.e., permanently damaged and initially survived but finally damaged, into the damaged category. For survived *boro* category, we considered the other two conditions, i.e., somehow survived with poor condition and survived/nor affected. The most affected district was Sunamgonj, where about 65% of its cultivated *boro* was damaged. The next affected district was Kishoregonj, where about 44% of the cultivated *boro* was damaged. Netrokona district showed about 21% damage of its cultivated *boro*.

## 5. Discussion

Once we compared our results of generating the Landsat-derived cultivated *boro* rice acreage (i.e., *r*^2^-value of 0.92), we found that similar results were also reported in the literature. For example: (i) Chen et al. [34] witnessed the accuracy around 76% in case of the Philippines; (ii) Mosleh et al. [23] attained accuracy in the range of 93 to 94% in case of Bangladesh; and (iii) Jin et al. [10] spotted the precision level around 85% in Sanjiang Plain in China. In addition, we also evaluated our results of estimating the MODIS-derived damaged *boro* rice acreage (i.e., *r*^2^-value of 0.92) against other relevant studies. In fact, we observed that similar results were found in other literature, such as: (i) Dao and Liou [17] demonstrated 2.52% difference with the community-level statistical data in the central region of Cambodia; (ii) Kwak et al. [18] showed about 80% consistency with the government census based on field-scale investigation and survey in Cambodia; and (iii) Chowdhury and Hassan [19] found 75% crop damage information was agreed with the government estimates in southwest Bangladesh.

As a result of the early flash flooding triggered on 27 March 2017, we employed the MODIS-derived NDVI time-series to map the dynamics of *boro* conditions. Our analysis revealed that four *boro-specific* conditions, i.e., (i) survived/not affected, (ii) initially survived but finally damaged, (iii) somehow survived but poor condition, and (iv) totally damaged *boro*. We found that the mean NDVI-values showed increasing trends (i.e., from lower NDVI-values to higher) from the initial planting/transplanting to mature stages during the pre-flash flooding event. Furthermore, we observed that these trends then emerged into four distinctive patterns of NDVI-values following the flooding event. For example: the “survived/not affected *boro*” class showed the temporal dynamics of the signatures similar to the natural gradual declining trend (i.e., from higher NDVI-values to lower) from the mature to harvesting stages of *boro* during April to May 2017. Such declining trend of the NDVI-values in the harvesting stage was due to the ripening process of the crop, when the mature green plants with rice seeds started to become golden or yellowish by losing its chlorophyll and water contents. Our observed temporal dynamics of the signatures in relation to the mature and harvesting stages were found to be similar to those reported in literature (e.g., [9,14,23]). In case of “initially survived but finally damaged” class, we found that the temporal dynamics of the signatures had some NDVI-values in the first imaging date after the flooding event (i.e., 7 April 2017), but the NDVI-values were close to zero or negative in the next imaging dates (i.e., 23 April 2017 and onwards). This indicated that those *boro* crops were initially survived, but ultimately submerged and damaged. For the “somehow survived but poor condition” class, we observed that the temporal dynamics of the signatures as of having lower NDVI-values (e.g., 0.4) in 7 April 2017, and continue with the similar NDVI-values for the following imaging dates. These lower NDVI-values were much lower compared to the natural declining NDVI-values in the harvesting stage, which indicated that those *boro* were in poor health. Finally, the “totally damaged” class of *boro* showed a sharp drop of the temporal signatures with NDVI-values (i.e., close to zero or negative) in the image acquired on 7 April 2017 (i.e., the first image after the flooding event), which indicated the crop was totally submerged and damaged.

In the case of evaluating the accuracy of both cultivated and damaged *boro* extents, we observed that our results were strong, i.e., *r*^2^ = 0.92 for both cultivated and damaged *boro* acreages. Despite such agreements, it might be possible to have some uncertainties in relation to our proposed classification schemas; which was potentially reduced as we used aggregated acreage information at district-level. In order to eliminate this sort of situation, detailed ground-based information would be highly recommended; this is beyond the scope of this study.

Among the six districts of interest, we observed that the districts of Sylhet and Maulavibazar had less cultivated *boro*. In fact, these districts have relatively higher elevated lands with a higher amount of rainfall, which makes the landscape more suitable for tea plantation. We also observed that the three districts of Sunamgonj, Kishoregonj, and Netrokona had experienced higher amounts of damaged *boro*. This happened due to the presence of higher percentages of *haor* and low lying areas in these districts. Additionally, Sylhet and Maulavibazar districts were less affected because of the presence of less *haors* in this portion of the study area.

## 6. Conclusions

In this study, we proposed a simple but comprehensive remote sensing-based mechanism for delineating the spatial extent of both cultivated and damaged *boro* areas due to a flash flooding over the *haor* basin in northeastern Bangladesh. We demonstrated the effectiveness of using Landsat-8 and MODIS based time-series for mapping cultivated and damaged *boro* acreages, respectively. Our results showed that a strong relationship existed in the event of comparing the outcomes with the ground-based estimates (i.e., *r*^2^ = 0.92 for both cultivated and damaged *boro* acreages). Despite the accuracy of our proposed methods, we would strongly recommend that evaluation of the proposed method should thoroughly be investigated prior to it being applied to other jurisdictions.

In addition, we suggest that Bangladesh should explore the applicability of adopting dry season rice varieties with a shorter growing season that spans 90 days, where the current varieties are of about 150 days. As such, the rice crop can be harvested prior to the initiation of the flash flooding season that is usually common in the month of April. Another option could be the analysis of climatic conditions, in particular temperature and precipitation regimes, to determine whether it is possible to start the *boro* season earlier than the current practices.
